# Uridine diphosphate N-acetylglucosamine orchestrates the interaction of GlmR with either YvcJ or GlmS in *Bacillus subtilis*

**DOI:** 10.1038/s41598-020-72854-2

**Published:** 2020-09-29

**Authors:** Elodie Foulquier, Frédérique Pompeo, Deborah Byrne, Henri-Pierre Fierobe, Anne Galinier

**Affiliations:** 1 Laboratoire de Chimie Bactérienne, UMR7283, Institut de Microbiologie de La Méditerranée, CNRS, Aix-Marseille Université, 31 Chemin Joseph Aiguier, 13402 Marseille Cedex 20, France; 2 Institut de Microbiologie de La Méditerranée, Protein Expression Facility, CNRS, Aix Marseille Université, 31 Chemin Joseph Aiguier, 13402 Marseille Cedex 20, France

**Keywords:** Biochemistry, Microbiology

## Abstract

In bacteria, glucosamine-6-phosphate (GlcN6P) synthase, GlmS, is an enzyme required for the synthesis of Uridine diphosphate N-acetylglucosamine (UDP-GlcNAc), a precursor of peptidoglycan. In *Bacillus subtilis*, an UDP-GlcNAc binding protein, GlmR (formerly YvcK), essential for growth on non-glycolytic carbon sources, has been proposed to stimulate GlmS activity; this activation could be antagonized by UDP-GlcNAc. Using purified proteins, we demonstrate that GlmR directly stimulates GlmS activity and the presence of UDP-GlcNAc (at concentrations above 0.1 mM) prevents this regulation. We also showed that YvcJ, whose gene is associated with *yvcK* (*glmR*), interacts with GlmR in an UDP-GlcNAc dependent manner. Strains producing GlmR variants unable to interact with YvcJ show decreased transformation efficiency similar to that of a *yvcJ* null mutant. We therefore propose that, depending on the intracellular concentration of UDP-GlcNAc, GlmR interacts with either YvcJ or GlmS. When UDP-GlcNAc concentration is high, this UDP-sugar binds to YvcJ and to GlmR, blocking the stimulation of GlmS activity and driving the interaction between GlmR and YvcJ to probably regulate the cellular role of the latter. When the UDP-GlcNAc level is low, GlmR does not interact with YvcJ and thus does not regulate its cellular role but interacts with GlmS to stimulate its activity.

## Introduction

The cell wall protects and shapes most bacteria (see^1–3^ for reviews). Its main component is the peptidoglycan (PG), a three-dimensional polymer that is continuously remodeled during growth and whose synthesis is also a prominent target of antibiotics. This mesh-like sacculus surrounds the cytoplasmic or inner membrane and is composed of glycan chains crosslinked by short peptides. PG precursors are synthesized in the cytoplasm and then exported across the cytoplasmic membrane, to be incorporated into pre-existing sacculus by the action of enzymes displaying either synthesizing or hydrolyzing activities.

One of these cytoplasmic precursors is Uridine diphosphate N-acetylglucosamine (UDP-GlcNAc)^4^. This nucleotide-sugar is synthesized via the hexosamine biosynthesis pathway. During the first step of this pathway, fructose 6-phosphate (F6P) and glutamine (Gln) are converted into glucosamine-6-phosphate (GlcN6P) and Glutamate^5^. This reaction is catalyzed by the rate-limiting GlcN6P synthase, GlmS. GlcN6P is subsequently converted into UDP-GlcNAc by three successive reactions involving the essential enzymes GlmM and GlmU^4,6^. GlmS is also essential, unless amino sugars are available in the environment^7–9^. Indeed, these amino sugars can be taken up and directly converted to GlcN6P, bypassing the reaction catalyzed by GlmS.

Bacteria possess sophisticated mechanisms to regulate UDP-GlcNAc homeostasis^10^. This regulation occurs at the first step of the hexosamine biosynthesis pathway and mainly acts on GlmS intracellular concentration, at the post-transcriptional level. Although the molecular mechanisms involved are different among bacteria, they aim to mediate feedback inhibition of *glmS* transcription by the product of its reaction, GlcN6P, to adjust GlmS concentration to the requirements of the cell. In the Gram-negative model bacterium *Escherichia coli*, two highly similar small RNAs, GlmY and GlmZ, and an RNase adaptor protein, RapZ that senses the GlcN6P level in the cell^11^ governs GlmS intracellular concentration^12,13^. RapZ is able to bind to these two RNAs, and despite their similarity, only GlmZ is able to activate the *glmS* transcript by base-pairing^12,13^. When GlcN6P level is high in the cell, RapZ bound to GlcN6P releases GlmY which is rapidly degraded^11^; GlmY is thus present at low amounts. RapZ binds GlmZ and facilitates the degradation of the latter by RNase E. Hence, the *glmS* transcript is not activated and not translated^12,13^. When GlcN6P level is low, RapZ is free and stimulates indirectly the expression of *glmY.* High levels of GlmY accumulates, binds and sequesters RapZ thus protecting GlmZ from degradation by RNaseE and promoting the expression of *glmS*^14^.

In the Gram-positive model bacterium *Bacillus subtilis*, this is a metabolite responsive ribozyme that controls GlmS concentration^15–17^. This well-studied ribozyme is a cis-regulatory RNA element located in the 5′ UTR of the *glmS* transcript that is able to bind GlcN6P. At low intracellular GlcN6P levels, the *glmS* ribozyme is inactive and *glmS* is transcripted. At high concentrations, GlcN6P binds to this cis-regulatory RNA element and activates self-cleavage. This activity generates a 5′-hydroxylated *glmS* transcript that is specifically recognized and degraded by RNase J1.

A recent publication proposed another level of GlmS regulation in *B. subtilis*^9^. Indeed, in this genetic study, the authors proposed that GlmS activity is stimulated by GlmR (formerly YvcK), a UDP-sugar binding protein^18^. This protein was previously shown to be essential for growth on non-glycolytic carbon sources like intermediates of the tricarboxylic acid cycle and substrates of pentose phosphate pathway, but dispensable for growth on glucose and other glycolytic carbon sources^18,19^. Substitutions affecting the UDP-sugar binding site of GlmR do not affect bacterial growth on these carbon sources^18^. It was thus proposed that binding of UDP-GlcNAc to GlmR may attenuate the stimulation of GlmS activity^9^. GlmR-stimulatory effect is probably essential during gluconeogenesis since F6P, the GlmS substrate, is present at low levels under these conditions.

Here, using purified proteins and measuring the production of GlcN6P, we showed that GlmR directly regulates GlmS activity and this stimulatory effect is inhibited by UDP-GlcNAc. We also observed that YvcJ, a protein homologous to RapZ and whose gene is associated with the *glmR* gene, binds to UDP-GlcNAc and interacts with GlmR only when GlmR is itself bound to UDP-GlcNAc. Furthermore, we observed that strains producing GlmR variants unable to interact with YvcJ have a similar reduction in transformation efficiency as a null *yvcJ* mutant. Altogether these results show that UDP-GlcNAc controls the cellular role of GlmR and its interaction with either YvcJ or GlmS. Indeed, at high concentrations, this UDP-sugar promotes the GlmR-YvcJ interaction and thus potentially regulates the cellular role of YvcJ. At low concentrations, it promotes the GlmR-GlmS interaction and thus the stimulation of GlmS activity.

## Results and discussion

### GlmS activity is directly stimulated by GlmR

In a recent study, Patel et al. suggested that GlmR activates GlmS in *B*. *subtilis*, probably directly since the two proteins interact by a bacterial two-hybrid assay^9^. To test this assumption we firstly decided to measure GlmS activity in the presence of increasing concentrations of GlmR, by an enzyme-coupled assay using yeast GlcN6P *N*-acetyltransferase 1, GNA-1^20^. For this purpose, we overproduced and purified these three proteins. Firstly, GNA-1 activity was tested by monitoring the presence of CoASH (Fig. S1). Then, GlmS activity was also tested (Fig. S2). Based on previous studies performed with GlmS from *E. coli*^20^ and when we compared the activity of GlmS from *B. subtilis* with that from *E. coli* (Fig. S3) we observed that the enzyme from *B. subtilis* has a weaker activity, lower than that from *E. coli*. Indeed, it probably requires stimulation by GlmR.

**Figure 1:**
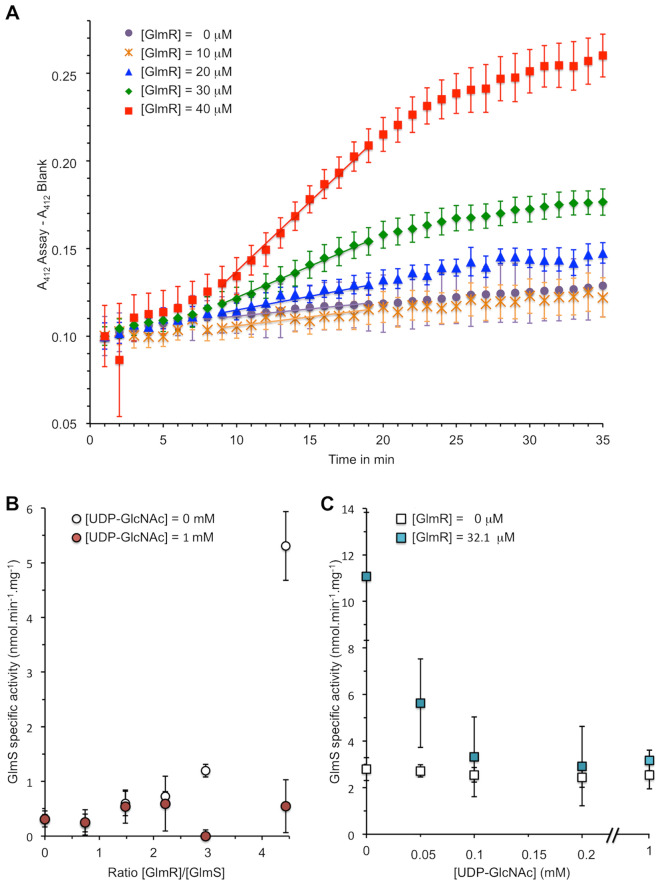
Measurement of GlmS activity in the presence or in the absence of GlmR and UDP-GlcNAc. Activity of GlmS from *B. subtilis* was measured by enzyme coupled assay. For each experiment, a reaction without GlmS was performed as negative control and used for background correction. Each experiment was reproduced at least in triplicate and error bars represent standard deviations. (**A**) Kinetic of GlmS activity in the presence of increasing concentration of GlmR. 7.3 µM of GlmS (48 µg in 100 µl) were incubated in the presence 0, 10, 20, 30 and 40 µM of GlmR in a final volume of 100 µl as indicated in the experimental procedures section. The amount of CoASH produced was monitored at 412 nm during 30 min by microplate reader at 37 °C as described previously^20^. We observed that the effect of GlmR on GlmS activity is optimal after 8 min of incubation; consequently, to calculate the amount of GlcN6P produced per min by GlmS, we measured the slope between 18 and 8 min. (**B**) GlmS activity in the presence of increasing amount of GlmR in the absence or in the presence of 1 mM UDP-GlcNAc. 7.3 µM of GlmS (48 µg in 100 µl) were incubated in the presence 0, 5.4, 10.7, 16.1, 21.4 and 32.1 µM of GlmR as indicated in the material and methods section. The amount of CoASH produced was monitored at 412 nm by microplate reader at 37 °C and the specific activity of GlmS was calculated as indicated in Figs. S1 and S2. (**C**) GlmS activity in the presence of increasing amount of UDP-GlcNAc and in the absence or in the presence of GlmR. 5.3 µM of GlmS (35 µg in 100 µl) were incubated in the absence or in the presence of 32.1 µM of GlmR ([GlmR]/[GlmS] = 6) and 0, 0.05, 0.1, 0.2, 0.5 and 1 mM of UDP-GlcNAc. The amount of CoASH produced was monitored at 412 nm by microplate reader at 37 °C and the specific activity of GlmS was calculated as indicated previously.

After determining the appropriate experimental conditions of the enzyme-coupled assay, the production of GlcN6P was measured in the presence of increasing amounts of GlmR (Fig. 1A). We observed that the addition of GlmR increases GlmS specific activity (Fig. 1B). Indeed, in the absence of GlmR, GlmS produced 0.32 nmoles min^−1^ mg^−1^ of GlcN6P in the experimental conditions tested. When 32.4 µM of GlmR was added to the sample (ratio [GlmR]/[GlmS] = 4.4), the production of GlcN6P was increased up to 5.31 nmoles min^−1^ mg^−1^ thereby indicating that, in these experimental conditions, GlmS specific activity was stimulated 16-fold by GlmR (Fig. 1B). This result clearly indicates that GlmR directly activates GlmS. This was confirmed by measuring directly GlmS activity via detection of GlcN6P produced by High Pressure Anion Exchange Chromatography coupled with Pulsed Amperometric Detection (HPAEC-PAD) (Fig. S4).

### Stimulation of GlmS by GlmR is antagonized by UDP-GlcNAc

Stimulation of GlmS activity was proposed to be antagonized when GlmR is bound to UDP-GlcNAc^9^. To test this assumption, we carried out the same experiment with an excess of UDP-GlcNAc (1 mM); the affinity of GlmR for UDP-GlcNAc was previously determined with an apparent *K*_D_ = 0.41 ± 0.24 mM^18^. In the presence of 1 mM of UDP-GlcNAc, as expected, we did not detect the GlmR-stimulatory effect on GlmS activity (Fig. 1B). To determine the amount of UDP-GlcNAc necessary to inhibit the GlmR booster effect, GlmS activity was also monitored in the absence and in the presence of GlmR ([GlmR]/[GlmS] = 6) incubated with increasing amount of UDP-GlcNAc (Fig. 1C). In such conditions, we have observed that in the absence of UDP-GlcNAc, GlmR stimulates GlmS activity by fourfold but the addition of 50 μM UDP-GlcNAc is sufficient to reduce twofold the stimulating effect of GlmR on GlmS activity. With only 100 μM of UDP-GlcNAc, the stimulatory effect of GlmR on GlmS activity is almost completely abolished.

### The yvcJ gene, encoding a RapZ homologue, is syntenic with glmR (yvcK)

**Figure 2:**
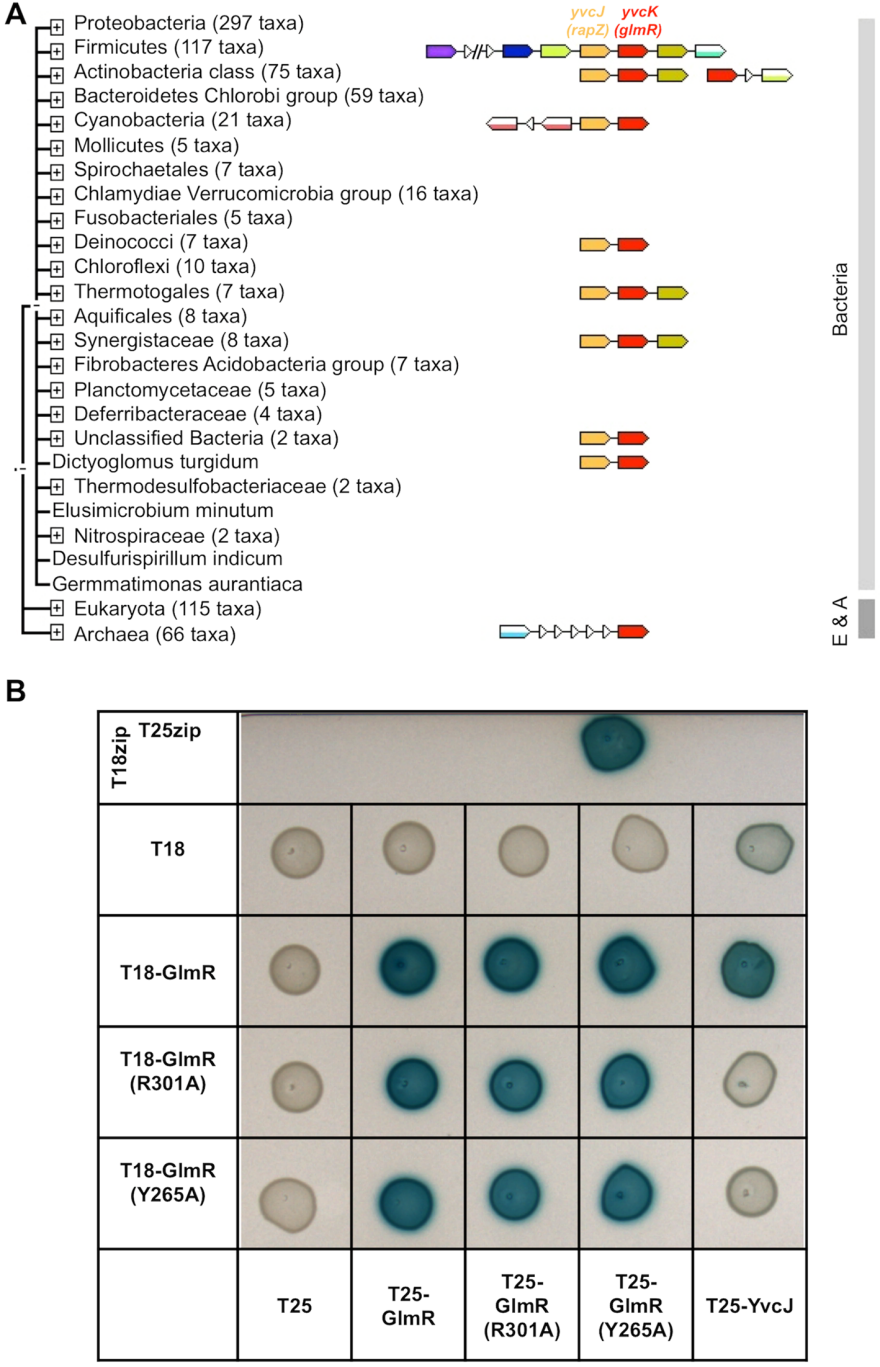
Genomic context of *glmR (yvcK*) and analysis of a potential interaction between GlmR and YvcJ by bacterial two hybrid. (**A**) Genomic context of *glmR* using Version 9.0 of STRING (https://string-db.org). Concerning Archaea, the gene homologous to *glmR* encodes CofD, a 2-phospho-lactate transferase that catalyzes the last step in the biosynthesis of coenzyme F(420). CofD is highly conserved among F(420)-producing organisms but possesses weak sequence homology with GlmR (or YvcK) found in non-F(420)-producing organisms^33^. In these non-F(420)-producing bacteria, *yvcK* (*glmR*) seems always associated to *yvcJ* (or *rapZ*). (**B**) Analysis of GlmR*,* GlmR(R301A), GlmR(Y265A) interactions with YvcJ by bacterial two hybrid assays. The T18 and T25 fragments of the adenylate cyclase protein were fused to GlmR*,* GlmR(R301A), GlmR(Y265A*)* and YvcJ. The pT18 and pT25 derivative plasmids were transformed into *E*. *coli* strain BTH101 that were spotted onto LB medium supplemented with X-Gal and IPTG and incubated at 30 °C overnight. When co-produced protein fusions interact, the *Bordetella pertussis* adenylate cyclase is active and the colonies are blue in the presence of X-Gal. Cells containing pT18 and pT25 empty vectors were used as negative control and cells containing pT18-ZIP and pT25-ZIP plasmids as positive control. Each experiment was reproduced at least in triplicate. Full-length picture of the petri dish is presented in Fig. S6.

When we analyzed the genetic context of the *glmR* (*yvcK*) locus, we observed that the *yvcJ* gene is immediately upstream of *glmR* (Fig. 2A). The role of *yvcJ* in *B. subtilis* is unclear, but interestingly, this gene is homologous to *rapZ,* the gene encoding the RNase adaptor protein which regulates GlmS concentration in *E. coli*^13,14^.

A biochemical characterization of YvcJ showed that it is a nucleotide binding protein with Walker A and B motifs, which exhibits NTPase and phosphatase activities^21^. The consensus sequence RxRxKNxQxRHRTxxKRK present in the C-terminal part of RapZ and known to specifically bind to RNA is not conserved in YvcJ homologs (Fig. S5 and^22^) and there is no evidence that *B. subtilis* YvcJ is a RNA-binding protein. In *B. subtilis,* deletion of the *yvcJ* gene or point mutation in the nucleotide-binding Walker A motif (replacement of catalytic K22 by A) reduces competence efficiency in comparison to a wild type strain^21,23^. However, an *yvcJ* deletion does not affect GlmS expression and, unlike the *glmR* deletion, does not induce a growth defect regardless of the carbon source present in the growth medium^19,21^.

The proximity of *yvcJ* and *glmR* genes in various genomes suggests that the corresponding proteins have a functional relationship. In addition, in their search, looking for mutations rescuing the growth defect of a *glmR* mutant, Patel et al. isolated a strain containing a missense mutation in *yvcJ;* they hypothesized an interaction between YvcJ with either GlmR or the *glmS* ribozyme^9^*.*

### GlmR interacts with YvcJ in an UDP-GlcNAc dependent manner

We therefore investigated a possible interaction between YvcJ and GlmR. For this purpose, we carried out bacterial two hybrid assays (Fig. 2B). We observed that, in fact, the two proteins interact with each other. We also carried out bacterial two hybrid assays with two modified forms of GlmR, GlmR(Y265A) and GlmR(R301A), which are affected in the UDP-sugar binding site and not capable of binding UDP-GlcNAc^18^. We observed that the interaction of YvcJ with the two modified forms of GlmR is strongly impaired suggesting that GlmR interacts with YvcJ only in the presence of UDP-GlcNAc (Fig. 2B).

**Figure 3:**
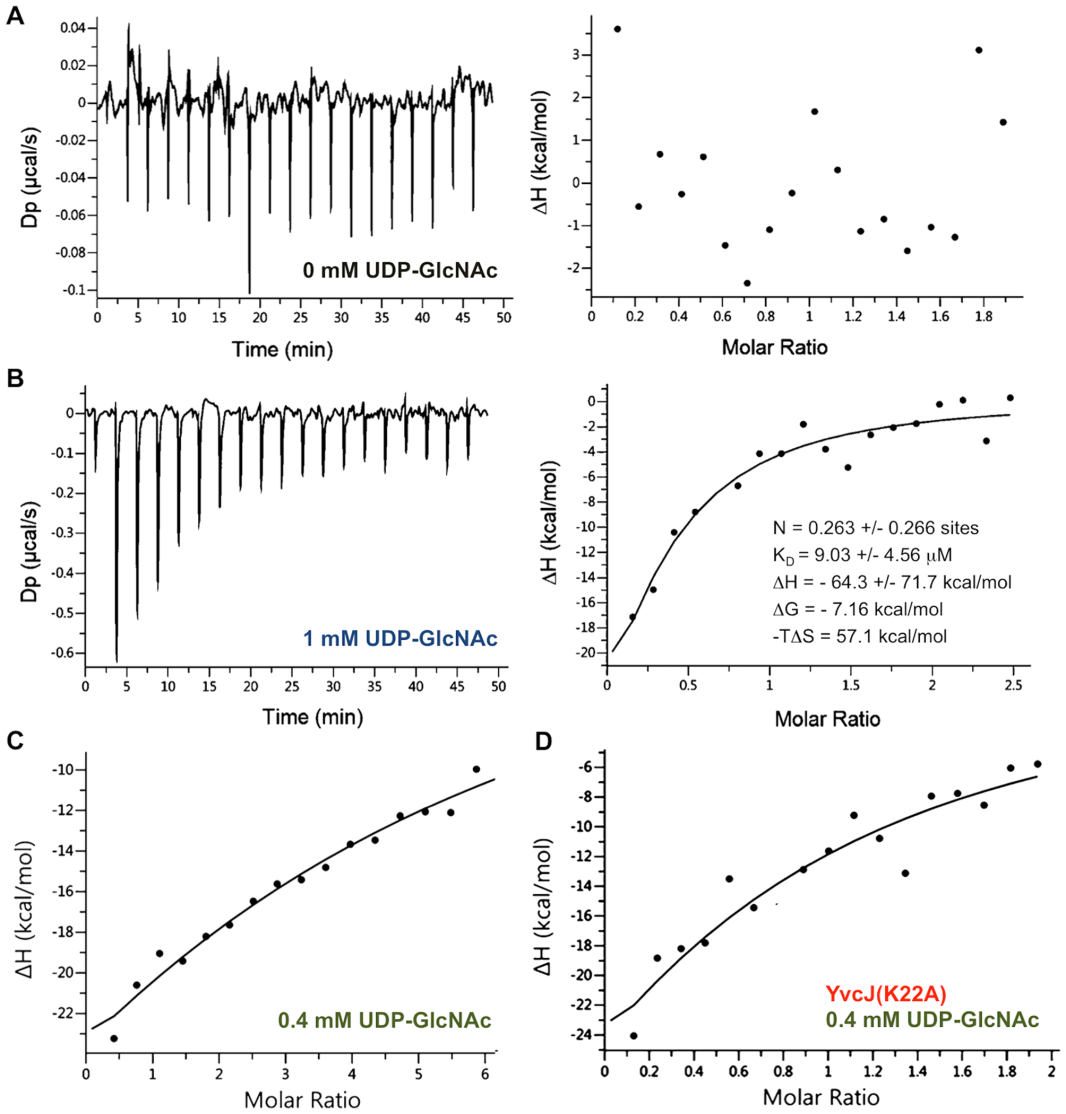
Analysis of the interaction of GlmR with YvcJ by ITC. For all the experiments, the reference experiment with the titrant protein injected into the cell containing buffer 50 mM Tris–HCl pH 7.5, 50 mM NaCl and 5% glycerol and 0, 0.4 or 1 mM UDP-GlcNAc was subtracted from the experimental data before analysis. Each experiment was reproduced at least in triplicate. (**A**) YvcJ in the absence of UDP-GlcNAc. (**B**) YvcJ in the presence of 1 mM UDP-GlcNAc. For these two experiments, the titrant protein (in the syringe) is GlmR at a concentration of 210 µM; it is injected into the sample cell containing 16 µM of YvcJ. The left panel shows heat exchange upon ligand titration and right panel shows the corresponding integrated data with binding isotherms fitted to a single–site binding model. (**C**) YvcJ in the presence of 0.4 mM UDP-GlcNAc. The titrant YvcJ (88 µM) was injected into a cell containing 2.5 µM GlmR at 37 °C in the presence of 0.4 mM UDP-GlcNAc. (**D**) YvcJ(K22A) in the presence of 0.4 mM UDP-GlcNAc. The titrant YvcJ(K22A) (109 µM) was injected into a cell containing 10 µM GlmR at 37 °C in the presence of 0.4 mM UDP-GlcNAc. For these two experiments, only the integrated data with binding isotherms fitted to a single–site binding model are presented. However, the interaction between YvcJ (WT or K22A) and GlmR is too weak to determine the thermodynamic parameters and the *K*_D_ in a reliable and accurate way.

To test this hypothesis, we explored the interaction between GlmR and YvcJ in the absence and in the presence of UDP-GlcNAc by using IsoThermal microCalorimetry (ITC). We failed to measure any detectable interaction between YvcJ and GlmR in the absence of UDP-GlcNAc (Fig. 3A). In contrast, in the presence of 1 mM of UDP-GlcNAc, we observed that each injection induced a large heat change (Fig. 3B), proportional to the complex formed^24^. The fitted isotherm yields the binding enthalpy ΔH, the equilibrium dissociation constant *K*_D_ and the stoichiometry n. From these data, the Gibbs free energy, ΔG and entropy were also calculated. The *K*_D_ calculated was 9.0+/− 4.6 µM and the thermodynamic parameters are shown in Fig. 3B. To determine whether the concentration of UDP-GlcNAc plays a role in the dissociation constant, the experiment was performed in the presence of 0.4 mM UDP-GlcNAc (Fig. 3C). In such conditions, we observed a weaker interaction between YvcJ and GlmR, showing that higher amounts of UDP-GlcNAc are needed for a better interaction and to determine the thermodynamic parameters and the *K*_D_. This result confirms that YvcJ interacts with GlmR in an UDP-GlcNAc dependent manner. We have also tested the interaction between GlmR with YvcJ(K22A) that is unable to bind and hydrolyze ATP and has similar results to those of the wild type YvcJ (Fig. 3D).

### Preventing the interaction GlmR-YvcJ affects competence efficiency

We observed that 0.1 mM of UDP-GlcNAc was sufficient to completely inhibit the stimulatory effect of GlmR on GlmS activity but this concentration of UDP-sugar was insufficient to induce the strongest interaction between GlmR and YvcJ. It is tempting to speculate that, UDP-GlcNAc acts at two different levels depending on its concentration. Indeed, at low concentration, UDP-GlcNAc binds to GlmR and reduces or prevents GlmS stimulation. A total inhibition is obtained for a concentration above 0.1 mM. At higher concentrations (greater than 0.4 mM), GlmR bound to UDP-GlcNAc interacts with YvcJ.

A deletion of *yvcJ* gene reduces competence efficiency in comparison to a wild type strain^21,23^. To test if GlmR could regulate the cellular function of YvcJ, we determined the transformation frequency of three different *glmR* mutant strains, expressing GlmR with a modified residue in the UDP-sugar binding site. Strain SG520^18^ expresses GlmR(T14A) which is able to bind UDP-GlcNAc, and is used as control, and strains SG522 and SG523^18^ express respectively GlmR(Y265A) and GlmR(R301A) both unable to bind UDP-GlcNAc and also unable to interact with YvcJ (see Fig. 2B). These three strains produce GlmR variants at the same level as the wild-type strain 168 produces GlmR^18^. We observed that the transformation frequency of the strain expressing GlmR(T14A) was not reduced in comparison to wild-type strain 168 (Table 1). By contrast, transformation frequencies for the two mutant strains expressing respectively GlmR(Y265A) and GlmR(R301A) unable to interact with YvcJ were respectively 3.4 and sevenfold lower than that of the wild type strain. For the *yvcJ* null mutant strain, the transformation frequency was reduced 8.8-fold compared to the wild-type strain (Table 1). This result indicates that mutations that prevent GlmR from interacting with YvcJ affect competence efficiency. It demonstrates not only that the interaction between YvcJ and GlmR occurs in *B. subtilis* cells but also that GlmR regulates cellular role of YvcJ.

**Table 1:** Transformation frequencies of wild-type, *yvcJ* and *glmR* mutant strains. The transformation frequency corresponds to the ratio between the number of transformants per milliliter and the number of cells per milliliter and is the average of three independent measurements.

Strains	References	Genotypes	Transformation frequencies*10^7^
168	Laboratory stock	*trpC2*	9.3+/− 1.3
SG91	^21^	*trpC2* Δ*yvcJ*::*cat*	1.1+/− 0.3
SG520	^18^	*trpC2, glmRT14A*	16.2+/− 2.3
SG522	^18^	*trpC2, glmRY265A*	2.7+/− 0.4
SG523	^18^	*trpC2, glmRR301A*	1.3+/− 0.3

### GlmR stabilizes YvcJ

**Figure 4:**
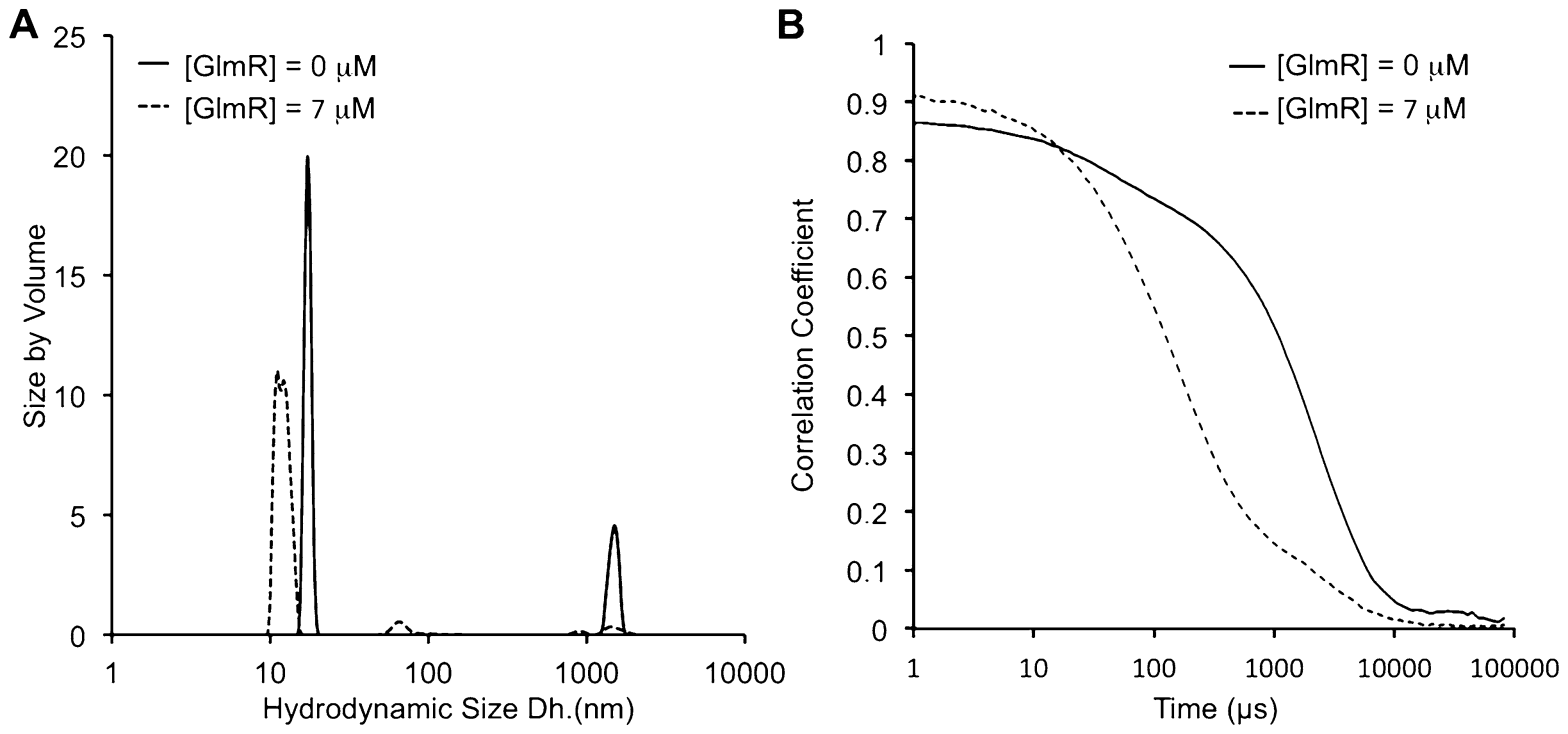
Analysis of the effect of GlmR on YvcJ by dynamic light scattering (DLS). (**A**) Volume weighted size distributions of 24 µM YvcJ in the presence of 0.4 mM UDP-GlcNAc and in the absence or in the presence of 7 µM GlmR at 25 °C using DLS. In black YvcJ is alone with the UDP-sugar. In black dashed line GlmR is added to YvcJ with a ratio of 1:0.3 YvcJ:GlmR. Three independent measurements were performed for each sample. (**B**) Real time correlation of intensity over time of YvcJ and GlmR in the presence of 0.4 mM UDP-GlcNAc average of triplicate. In black YvcJ is alone with the UDP-sugar and in black dashed GlmR is added to YvcJ with a ratio of 1:0.3 YvcJ:GlmR.

To analyze the stimulatory effect of GlmR on YvcJ from a biochemical point of view, we decided to analyze the effect of GlmR on the YvcJ oligomerization state by dynamic light scattering (DLS). As a control, first we analyzed YvcJ or GlmR alone in the absence and in the presence of 0.4 mM of UDP-GlcNAc. In the absence of UDP-GlcNAc, YvcJ shows the presence of large aggregates at 700 nm and an oligomeric complex of 12.6 nm (Fig. S7). When UDP-GlcNAc is added to YvcJ, the oligomeric shape is increased to 17 nm and large diffusing molecules with an average size of 1241 nm are apparent. YvcJ is polydispersed with two major populations that are a mix of aggregates and a large oligomer of approximately 15 molecules (Figs. 4A and S7). In addition, when YvcJ is alone with UDP-GlcNAc, we can observe the slow movement of large molecules over time (Fig. 4B). GlmR, in the absence of UDP-GlcNAc, has an average diameter size of 46 nm but in the presence of UDP-GlcNAc, GlmR is a mix of large diffusing molecules, with an average diameter size of 409 nM (Fig. S7); thus for both YvcJ and GlmR independently, the addition of UDP-GlcNAc leads to an increase in their oligomeric shape and induces aggregates.

On the other hand, when we titrate YvcJ with GlmR at a ratio 1:0.3 in the presence of 0.4 mM UDP-GlcNAc, conditions in which an interaction between GlmR and YvcJ occurs, we observed that the hydrodynamic size of YvcJ is reduced. In addition, we can observe an increase in the pace of the molecules, suggesting smaller molecules, and thus the dissolution of aggregates (Fig. 4B). The average molecule size reduces from 1241 to 124 nm (Fig. S7). Indeed, the large oligomer of YvcJ is reduced from 17 nm (15 molecules) to 12 nm (5 molecules). In the absence of UDP-GlcNAc, in such conditions no interaction between YvcJ and GlmR is detected by ITC (Fig. 3A), small amounts of aggregates of YvcJ are still vizualized by DLS and the oligomeric complex is larger at 15 nm (Fig. S7). We conclude that when GlmR is bound to YvcJ in the presence of UDP-GlcNAc, we observe a stabilizing effect of GlmR on YvcJ and a dissolution of the YvcJ aggregates.

### YvcJ also binds UDP-GlcNAc

**Figure 5:**
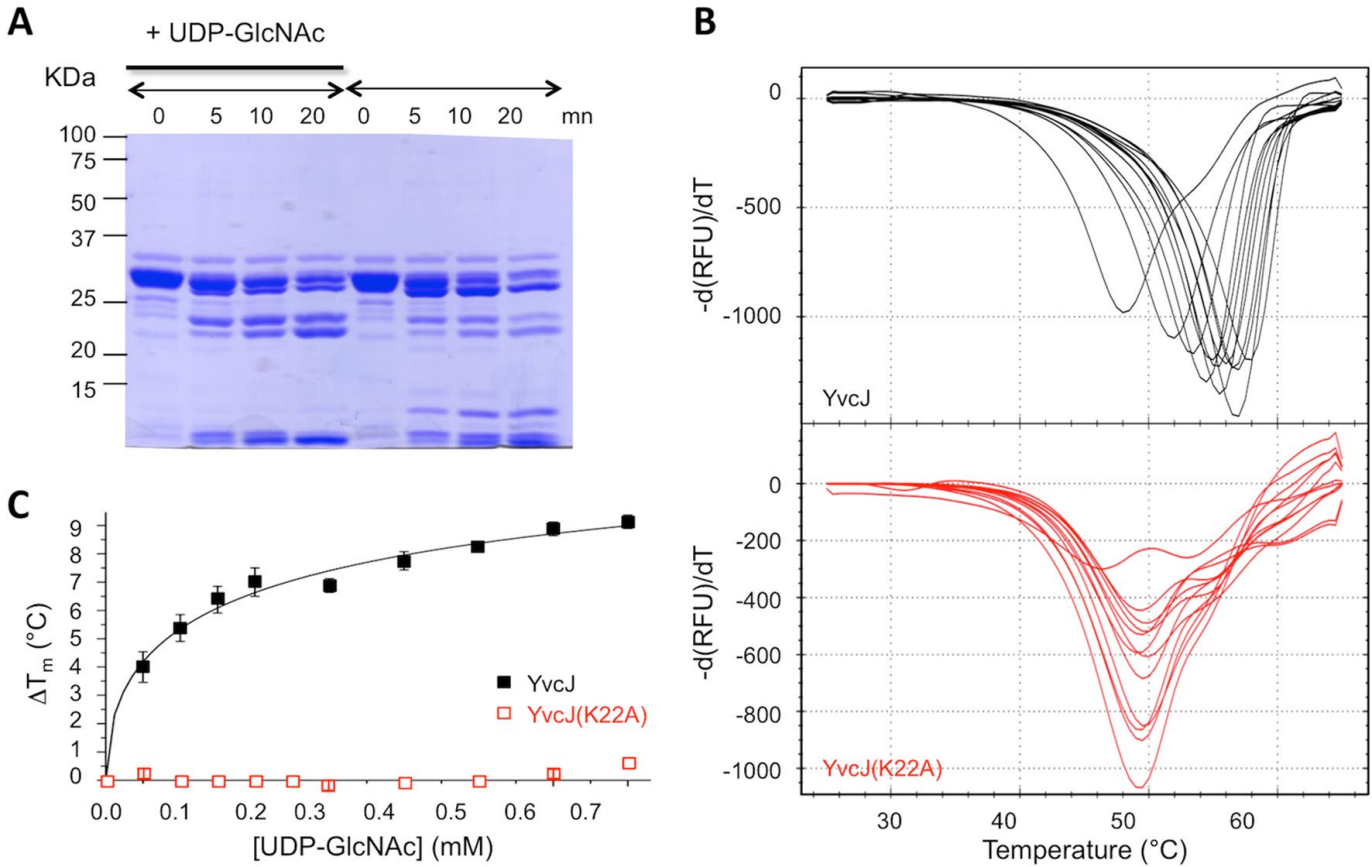
Investigation of the potential binding of UDP-GlcNAc to YvcJ by partial proteolysis and Thermal Shift Assay (TSA). (**A**) Coomassie-stained SDS-PAGE of YvcJ partial proteolysis profile. YvcJ was incubated with endoproteinase Glu-C (Promega) in the presence or in the absence of 1 mM UDP-GlcNAc for 0, 5, 10 or 20 min at 37 °C. The digestion profiles were assessed by electrophoresis in 12.5% SDS-PAGE. Full-length gel is presented in Fig. S8. (**B**) TSA in the presence of increasing concentrations of UDP-GlcNAc. YvcJ (top) and YvcJ(K22A) (bottom) melting profiles were monitored in the presence of increasing concentration of UDP-GlcNAc (0–1 mM). One curve corresponds to data obtained for one concentration of UDP-GlcNAc. The melting temperature of the protein (T_m_) is obtained at the midpoint of each melting curve and corresponds to the minimum of the negative derivative curves. The T_m_ is an indicator of protein stability and is increased by the addition of UDP-GlcNAc. For the WT protein, T_m_ = 48 °C in the absence of UDP-GlcNAc and T_m_ = 58.5 °C in the presence of 1 mM UDP-GlcNAc. For YvcJ(K22A), T_m_ = 49.5 °C and is not increased by addition of UDP-GlcNAc. (**C**) TSA results for the binding of UDP-GlcNAc to YvcJ. Assays were performed with YvcJ and YvcJ(K22A) in the presence of increasing concentrations of ligands (0–0.7 mM). The difference of temperature (the shift of T_m_ induced by the presence of ligand) was plotted against the concentration of UDP-GlcNAc. Each experiment was reproduced at least in triplicate and the standard deviations are represented by the error bars. Curve fitting was performed by using Microcal Origin 5.0 software (Microcal software Inc).

RapZ was recently shown to bind to GlcN6P^11^. During our DLS analysis, we observed an increase in the oligomeric shape of YvcJ when it was incubated alone with UDP-GlcNAc (see Fig. S7); this increase was no longer observed for YvcJ(K22A). We thus decided to test whether YvcJ was able to bind UDP-GlcNAc (structures of GlcN6P and UDP-GlcNAc possess similarities). For this purpose we used two independent techniques. First, we assessed YvcJ for sensitivity to limited proteinase digestion in vitro. As shown in Fig. 5A, the presence of UDP-GlcNAc weakly modifies YvcJ endoproteinase Glu-C sensitivity. This result suggests a direct binding of UDP-GlcNAc to YvcJ. This binding probably induces YvcJ conformational changes thus modifying the accessibility of endo Glu-C cleavage sites.

Secondly, to probe for other evidence of interaction, we used the Thermal Shift Assay (TSA) to known to determine apparent *K*_D_ of protein–ligand interactions (for example see^25,26^). By monitoring SYPRO Orange dye fluorescence in microplates using a thermal cycler, we have quantified the effects of UDP-GlcNAc on the thermal denaturation temperature of YvcJ (Fig. 5B). We observed that UDP-GlcNAc raises the melting temperature (T_m_) of YvcJ in a concentration-dependent manner. Moreover, the increases of T_m_ (of up to 10 °C) indicate that YvcJ binds to UDP-GlcNAc with an apparent *K*_D_ = 0.28 ± 0.47 mM (Fig. 5C). This value is in the same range for GlmR that was previously shown to bind to UDP-GlcNAc by TSA with an apparent *K*_D_ = 0.41 ± 0.24 mM^18^ but with a lower affinity compared with the *K*_D_ value of RapZ for GlcN6P (apparent *K*_D_ = 186 nM)^11^.

In addition, we carried out the same experiment with purified YvcJ(K22A) and no variation of T_m_ was observed and thus no binding to UDP-GlcNAc was monitored (Fig. 5B). Moreover, this inability of YvcJ(K22A) to bind to UDP-GlcNAc indicates that this UDP-sugar is not the key component for YvcJ binding to GlmR but rather the key component for GlmR binding to YvcJ (see Fig. 3D).

### The triad of proteins GlmS/GlmR/YvcJ: a “ménage à trois” in *B. subtilis* cells

In bacteria, UDP-GlcNAc is a key metabolite and its synthesis via the hexosamine pathway is highly regulated^10^. GlmS is the crucial branch-point enzyme that diverts F6P from glycolysis (carbon metabolism) to the hexosamine pathway (PG synthesis) and catalyzes the reversible conversion of F6P and Gln into GlcN6P and Glutamate. In *B. subtilis*, the product of the reaction, GlcN6P, binds to the *glmS* ribozyme to cleave the mRNA and suppresses translation of GlmS. In this study, we demonstrated that the downstream metabolite UDP-GlcNAc plays also a key role in GlmS regulation. Indeed, we showed that GlmR directly activates GlmS and this stimulation is antagonized when GlmR is bound to UDP-GlcNAc. Equally, UDP-GlcNAc drives an interaction between GlmR and YvcJ to stabilize YvcJ and regulate its cellular role.

**Figure 6:**
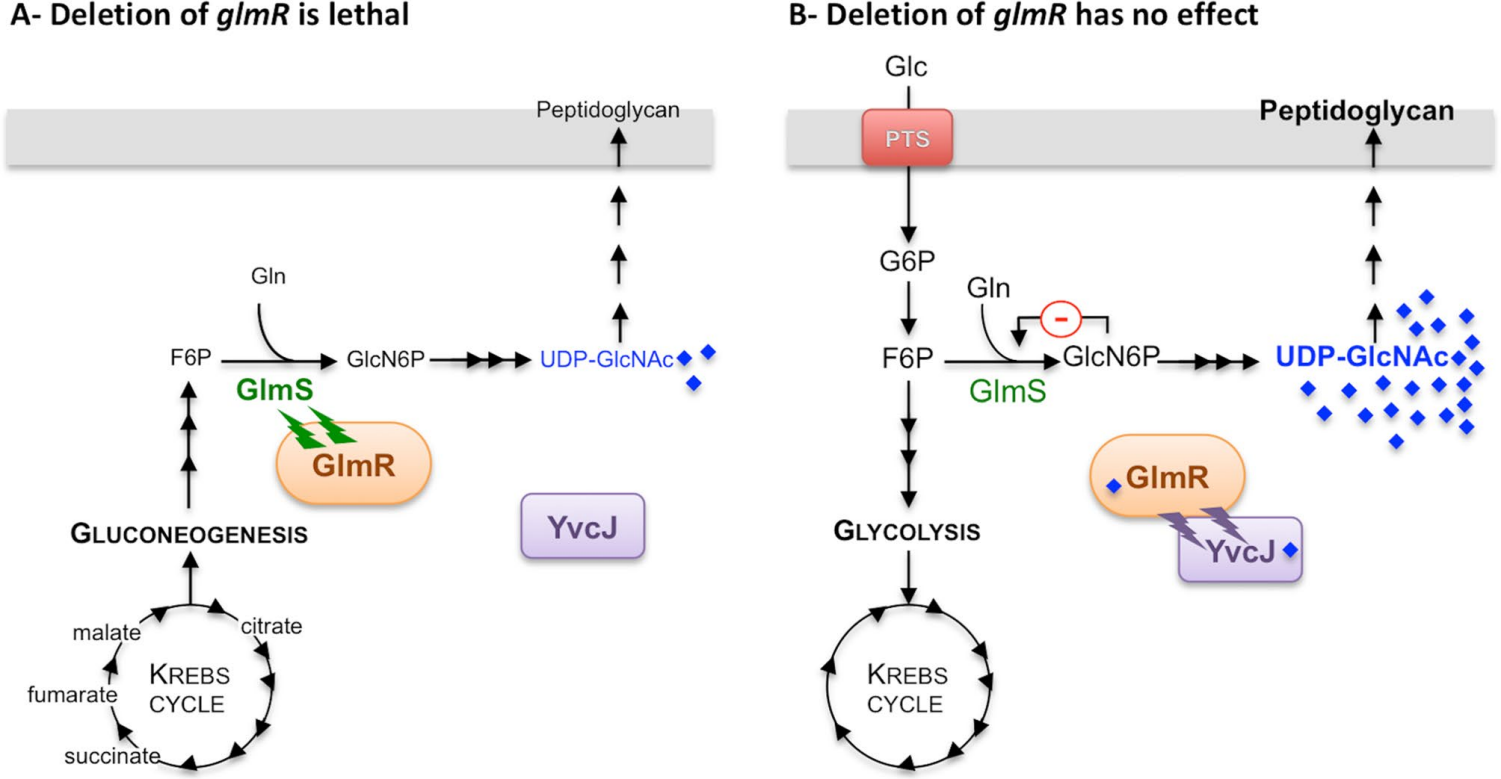
Schematic representation of GlmS regulation by GlmR, UDP-GlcNAc and YvcJ in *B. subtilis* depending on growth conditions. (**A**) When *B. subtilis* is grown in the presence of non-glycolytic carbon sources like intermediates of Krebs cycle, the *glmR* mutant cells have an abnormal rod-shape then lyse and the deletion of *glmR* is lethal^19^. In such conditions, stimulation of GlmS by GlmR is essential for a sufficient production of UDP-GlcNAc and thus for a correct PG synthesis; YvcJ is free. (**B**) When *B. subtilis* is grown in the presence of glycolytic carbon sources like glucose, the *glmR* mutant cells have a normal rod-shape and deletion of *glmR* has no effect^19^. The intracellular concentration of F6P is high, 16-fold higher in comparison to growth on malate^27^. Consequently, intracellular concentration of UDP-GlcNAc is probably high and therefore stimulation of GlmS by GlmR is not essential for correct synthesis of PG. In such conditions, GlmR is bound to UDP-GlcNAc and GlmS activity is not stimulated to avoid an excess of UDP-GlcNAc synthesis. In addition, GlmR bound to UDP-GlcNAc interacts with YvcJ to stabilize it and potentially stimulate its activity. In parallel, GlcN6P binds to *glmS* ribozyme and regulates GlmS intracellular concentration.

When *B. subtilis* is grown on non-glycolytic carbon sources, GlmR stimulates GlmS activity and this stimulation is essential for correct synthesis of PG. In such conditions, deletion of *glmR* induced morphologic abnormalities, including bulging cells before lysis^19^. This is possibly due to a deficit of UDP-GlcNAc in the *glmR* mutant and thus to an abnormal PG synthesis; the PG sacculus cannot maintain cell shape and bacterial integrity; this is consistent with the requirement for GlmR. In these conditions, YvcJ does not interact with GlmR. When *B. subtilis* is grown on glycolytic carbon sources, the intracellular concentration of F6P is high. Indeed, a metabolomic study indicates that, in *B. subtilis,* F6P intracellular concentration is about 16-fold higher during growth on glucose (glycolytic carbon source) than during growth on malate (gluconeogenic carbon sources)^27^. In glycolytic growth conditions, stimulation of GlmS by GlmR is not required for proper synthesis of PG. The *glmR* mutant cells have a normal rod-shape^19^. This indicates that, in the absence of GlmR, the intracellular concentration of UDP-GlcNAc is sufficient for a normal PG synthesis. In a wild type background, in the presence of high levels of UDP-GlcNAc, the sugar binds to GlmR, which can no longer stimulate GlmS activity. In such conditions, GlmR interacts with YvcJ to potentially stimulate its activity (Fig. 6). As a result, depending on the UDP-GlcNAc concentration, GlmR might have a booster effect either on the activity of GlmS or on that of YvcJ.

To date, the cellular role of YvcJ in *B. subtilis* is poorly characterized. YvcJ regulates neither the intracellular concentration of GlmS^21^ nor its activity since YvcJ interacts only with GlmR bound to UDP-GlcNAc that is unable to stimulate GlmS. In addition, UDP-GlcNAc, at concentrations below 0.1 mM, is insufficient to promote efficiently the GlmR-YvcJ interaction, but sufficient to antagonize GlmR booster effect on GlmS activity. Inactivation of *yvcJ* affects competence but the molecular mechanism whereby it influences competence is of yet unknown. There is no evidence that YvcJ is a RNA-binding protein since the sequence of RapZ that specifically binds to RNAs is not conserved in YvcJ^22^.

In conclusion, our work supports a model of the stimulation of GlmS activity upon interaction with GlmR as suggested previously^9^ and demonstrates an additional interaction between GlmR and YvcJ, a protein homologous to RapZ that regulates GlmS intracellular concentration in *E. coli* (Fig. 6). In the future, the characterization of the role of YvcJ in *B. subtilis* cells and the role of GlmR in *E. coli* may give clues concerning the triad formed by YvcJ, GlmR and GlmS proteins and this UDP-GlcNAc-dependent regulatory device for PG synthesis adapted to nutrient availability.

## Materials and methods

### Plasmids and strains construction

Standard procedures for molecular cloning and cell transformation were used. All the plasmids and primers used in this study are listed in Table 2. All the PCR-derived DNA fragments in the plasmid constructs were verified by sequencing (Eurofins Genomics). To produce the GlmS with a C-terminal 6His-tag, *glmS* gene was amplified by PCR from chromosomal DNA from *B. subtilis* 168 strain using GlmS-deb-Bam and GlmS-fin-Xho primers and introduced into the pET21a (+) plasmid (Novagen) between the BamHI and XhoI sites*.* The obtained recombinant plasmids were introduced into *E. coli* NiCo21(DE3) to avoid contamination with untagged *E. coli* GlmS during purification on Ni–NTA column^28^. For the generation of fusion proteins for the adenylate cyclase-based two-hybrid assay, *yvcJ* gene was amplified by PCR from *B. subtilis* 168 chromosomal DNA using T25yvcj-5 and T25yvcj-3 primers and inserted between the PstI and BamHI sites in pT25.

**Table 2:** List of plasmids and primers used in this study.

Plasmids	Primers	Sequences 5′–> 3′	References
pET21-YvcJ	YvcJ 5′	GTCAGGGGGGGGGATCCATGAGTGTTAGTG	^21^
	YvcJ 3′	TTTTGTCCCCTCGAGTTTCCGGCTTCT	
pET21-YvcJ(K22A)	yvcJK22A5'	GGAATGTCGGGAGCGGGGGCAACTGTCGCGATCCAAAGC	^21^
	yvcJK22A3'	GCTTTGGATCGCGACAGTTGCCCCCGCTCCCGACATTCC	
pQE30-GlmR	GlmR-pQE30-fin	TTCAAGGCTCTGCAGTCATTCTTTCAGT	^29^
	GlmR-pQE30-deb	AGAAGCCGGAGGATCCATGGGACAAA	
pET21-GlmS	GlmS-deb-Bam	AGGAAGGGATCCATGTGTGGAATCGTAGGTTA	This work
	GlmS-fin-Xho	GTTAAACTCGAGCTCCACAGTAACACTCTTCG	
pQE30-GNA-1			^32^
pT25 and pT25-Zip			^30^
pT18 and pT18-zip			^30^
pT25-YvcJ	T25yvcj-5	CGGGCTGCAGGGAGTGTTAGTGAATCACATG	This work
	T25yvcj-3	TACCCGGGGATCCTCTTATTTCCGGCTTCTCTTTTC	
pT18-GlmR	GlmR-T18-deb	CGGGTACCGATGGGACAAAAGCCG	^29^
	GlmR-T18-fin	CAAGCTTTCTTTCAGTAAATCAAC	
pT25-GlmR	GlmR-T25-deb	GGGCTGCAGGACAAAAGCCGAAAATC	^29^
	GlmR-T25-fin	GCGGATCCTCATTCTTTCAGTAAATCAAC	
pT18-GlmR (R300A) and pT25-GlmR (R300A)	R300AglmR-5	ACGTATAAAAATGACGTAATAGCTCACGATACACATAAAGTGGCC	This work
	R300AglmR-3	GGCCACTTTATGTGTATCGTGAGCTATTACGTCATTTTTATACGT	
pT18-GlmR (Y264A) and pT25-GlmR (Y264A)	Y264AglmR-5	CCCGACGAAATAAAACGTAAGGCCGAAATGGAATCGGCGCGTCCT	This work
	Y264AglmR-3	AGGACGCGCCGATTCCATTTCGGCCTTACG-TTTTATTTCGTCGGG	

### Site-directed Mutagenesis

The two point mutations were introduced into the gene by PCR amplification of the whole pT18-GlmR and pT25-GlmR plasmid, and for each plasmid, with a pair of primers with complimentary sequences: (R300AglmR-5; R300AglmR-3) to replace Arg300 by Ala and (Y264AglmR-5; Y264AglmR-3) to replace His264 by Ala. The primers were designed with mismatching nucleotides at their center and contain the mutation. Then, the PCR products were incubated at 37 °C for 2–3 h with 1 µl of DpnI that digests methylated parental strands, and then transformed into *E. coli*. The resulting constructs were verified by DNA sequencing.

### Protein purification

Purification of 6His-tagged recombinant proteins was performed with Ni^2+^-NTA resin (Qiagen, Hilden, Germany) as previously described^21,29^. Imidazole was removed using a PD-10 Column (GE Healthcare, Pittsburgh, PA) and all the proteins were stocked at − 80 °C in a buffer containing 50 mM Tris–HCl pH7.5, 50 mM NaCl, 5% Glycerol.

### GlmS activity assays via enzyme-coupled assay using GNA-1^20^

A typical assay solution contained 50 mM Tris–HCl pH 7.4, 1 mM EDTA, GlmS (0–7.3 µM), 2 mM F6P, 2 mM L-Gln, 0.5 mM Ac-CoA, 0.5 mM DTNB, 10 µg GNA-1 in a final volume of 100 µl. When indicated, GlmR (0–40 µM) and UDP-GlcNAc (0–1 mM) were added to the enzymatic tests. The amount of CoA produced was monitored at 412 nm by a microplate reader at 37 °C. For each experiment, a blank reaction without GlmS was performed as a negative control and used for background correction. All the experiments were carried out at least in triplicate. To calculate the amount of GlcN6P produced per min by GlmS in 100 µl, the slope between 18 and 8 min was measured; the number of pmoles of GlcN6P produced per min corresponds to (A_412 nm_ X 10,000)/2.9392 (see Fig. S1).

### Bacterial two-hybrid assays

The YvcJ and GlmR proteins were fused to the T18 or T25 catalytic domain of adenylate cyclase using plasmids pT18 and pT25^30^. Co-transformed strains of *E. coli* BTH101 expressing pT18-derivative plasmids and pT25-derivative plasmids were plated on LB agar and incubated at 30 °C for 48 h using the same protocol described in^29,31^. One milliliter of LB medium supplemented with 100 μg/ml ampicillin, 50 μg/ml chloramphenicol, and 0.5 mM IPTG was inoculated and incubated overnight at 30 °C. Ten microliters of the overnight culture were spotted on the LB medium plates containing appropriate antibiotics, 0.5 mM IPTG, and 40 μg/ml X-Gal. The plates were incubated overnight at 30 °C.

### Isothermal Titration Calorimetry (ITC)

Thermo-dynamic parameters were estimated by isothermal titration calorimetry (ITC) using a MicroCal PEAQ-ITC (Malvern, Malvern, UK) microcalorimeter. The working buffer for YvcJ and GlmR was 50 mM Tris–HCl pH 7.5, 50 mM NaCl and 5% glycerol. The experiments were carried out at 37 °C in the absence or in the presence of UDP-GlcNAc (0.4 or 1 mM) with 19 injections, first with an initial injection of 0.4 µl followed by 18 injections of 2 µl. The reaction was performed with a constant stirring speed of 750 rpm, each injection lasted 4 s with a 150-s interval between each injection. Reference measurements for titrant injected into buffer were subtracted from raw data. The data were fitted using a ‘One Set of Sites’ model in the (PEAQ-ITC Analysis Software). Each experiment was reproduced at least in triplicate.

### Measurement of transformation frequencies

One freshly streaked colony was inoculated into 1 ml of MC medium (100 mM Potassium Phosphate pH 7, 3 mM sodium citrate, 2% glucose, 22 mg/ml Ferric ammonium citrate, 0.1% casein hydrolysate and 0.2% Potassium glutamate) supplemented with 6 mM MgSO_4_ and 20 µg/ml tryptophan at 37 °C with shaking. After 4 h, 1.2 μg of a chromosomal DNA carrying a spectinomycin marker was added to 200 µl of bacteria. After an incubation for 2 h at 37 °C, bacteria were plated on LB medium in the presence and in the absence of antibiotic and incubated overnight at 37 °C. Transformation frequency was expressed as the ratio between the number of transformants ml^−1^ and the number of cells ml^−1^.

### Dynamic Light Scattering (DLS)

Dynamic light scattering (DLS) experiments were performed to complement the ITC interaction results of YvcJ:GlmR. This technique uses a light scattering technique, based on intensity and movement of a molecule in solution to determine its hydrodynamic size^18^. We used the Zetasizer Nano ZS from Malvern Instruments. The experiments were performed at 25 °C in disposable micro-cuvettes (ZEN0040 Malvern) with a final volume of 50 µl. Each protein solution was measured in triplicate, with approximately 15 runs for each measurement, the average protein size for 15 runs was calculated for each experiment. 24 µM of YvcJ was analyzed at 25 °C in the absence and presence of GlmR in buffer 50 mM Tris–HCl, pH 7.5, 50 mM NaCl, 5% glycerol, supplemented with 0.4 mM UDP-GlcNAc.

### Limited proteolysis

For each 20 μl sample, 6 µg of YvcJ were pre-incubated for 10 min at 37 °C with 40 mM NaCl, 1 mM MgCl_2_, 10 mM Tris/HCl, pH 7.5 in the absence or in the presence of UDP-GlcNAc (1 mM). After addition of 0.6 µg of Endoproteinase GluC (Promega), the reaction mixture was incubated for 0, 5, 10 or 20 min at 37 °C. The digestion was stopped by adding an equal volume of electrophoresis loading buffer to the assay mixtures and by heating 5 min at 100 °C. Then, the samples were analyzed by 12.5% sodium dodecyl sulfate polyacrylamide gel electrophoresis.

### Thermal Shift Assay (TSA)

In thin-walled 96-well PCR plates, each well (20 μl) contained 10 μM of YvcJ or YvcJ(K22A) and 2 μl of the fluorescent SYPRO Orange dye solution (Molecular Probes, 5000X, diluted to 100X in water), in 50 mM NaCl, 1 mM MgCl_2_, 5% glycerol, 50 mM Tris–HCl pH 7.5 in the presence of increasing concentrations of UDP-GlcNAc. The samples were heated from 25 to 65 °C in a real-time PCR apparatus CFX96 (Bio-Rad). The fluorescence intensity (Ex/Em = 470/570 nm) of SYPRO Orange was monitored and analyzed from the melt peak using CFX Manager software (Bio-Rad) as indicated in^18^. The shift of denaturation temperature (ΔT_m_) was plotted against the concentration of UDP-GlcNAc. Curve fitting was performed by using Microcal Origin 5.0 software (Microcal software Inc) using the following equation y = ΔT_m_ * x^n^/(apparent K_D_^n^ + x^n^), were n is the cooperative binding site. Each experiment was reproduced at least in triplicate.

## Supplementary information

Supplementary Information.

## Abbreviations

GlcN6P: Glucosamine-6-phosphate
PG: Peptidoglycan
UDP-GlcNAc: Uridine diphosphate N-acetylglucosamine
F6P: Fructose 6-phosphate
Gln: Glutamine
GlcN6P: Glucosamine-6-phosphate
GNA-1: GlcN6P *N*-acetyltransferase 1
HPAEC-PAD: High Pressure Anion Exchange Chromatography coupled with Pulsed Amperometric Detection
CoASH: Co enzyme A
ITC: IsoThermal microCalorimetry
DLS: Dynamic light scattering
TSA: Thermal Shift Assay
DTNB: 5,5-Dithiobis 2-nitrobenzoic acid
