# Supplementary material for: Uridine diphosphate N-acetylglucosamine orchestrates the interaction of GlmR with either YvcJ or GlmS in Bacillus subtilis
Source: Sci Rep. 2020 Sep 29;10:15938. doi: 10.1038/s41598-020-72854-2 (PMC7525490; doi:10.1038/s41598-020-72854-2)
Supplement: Supplementary file 1 — Supplementary Information. [file 41598_2020_72854_MOESM1_ESM.pdf]

Uridine diphosphate N-acetylglucosamine orchestrates the interaction of GlmR with either YvcJ or GlmS in *Bacillus subtilis*

Supplemental data

Elodie Foulquier[1], Frédérique Pompeo[1], Deborah Byrne[2], Henri-Pierre Fierobe[1] and Anne Galinier[1*]

[1] Laboratoire de Chimie Bactérienne, UMR7283, CNRS, Aix-Marseille Université, Institut de Microbiologie de la Méditerranée, 31 Chemin Joseph Aiguier 13402 Marseille cedex 20, France.

[2] Institut de Microbiologie de la Méditerranée, Protein Expression Facility, CNRS, Aix Marseille Université, 31 Chemin Joseph Aiguier 13402 Marseille cedex 20, France.

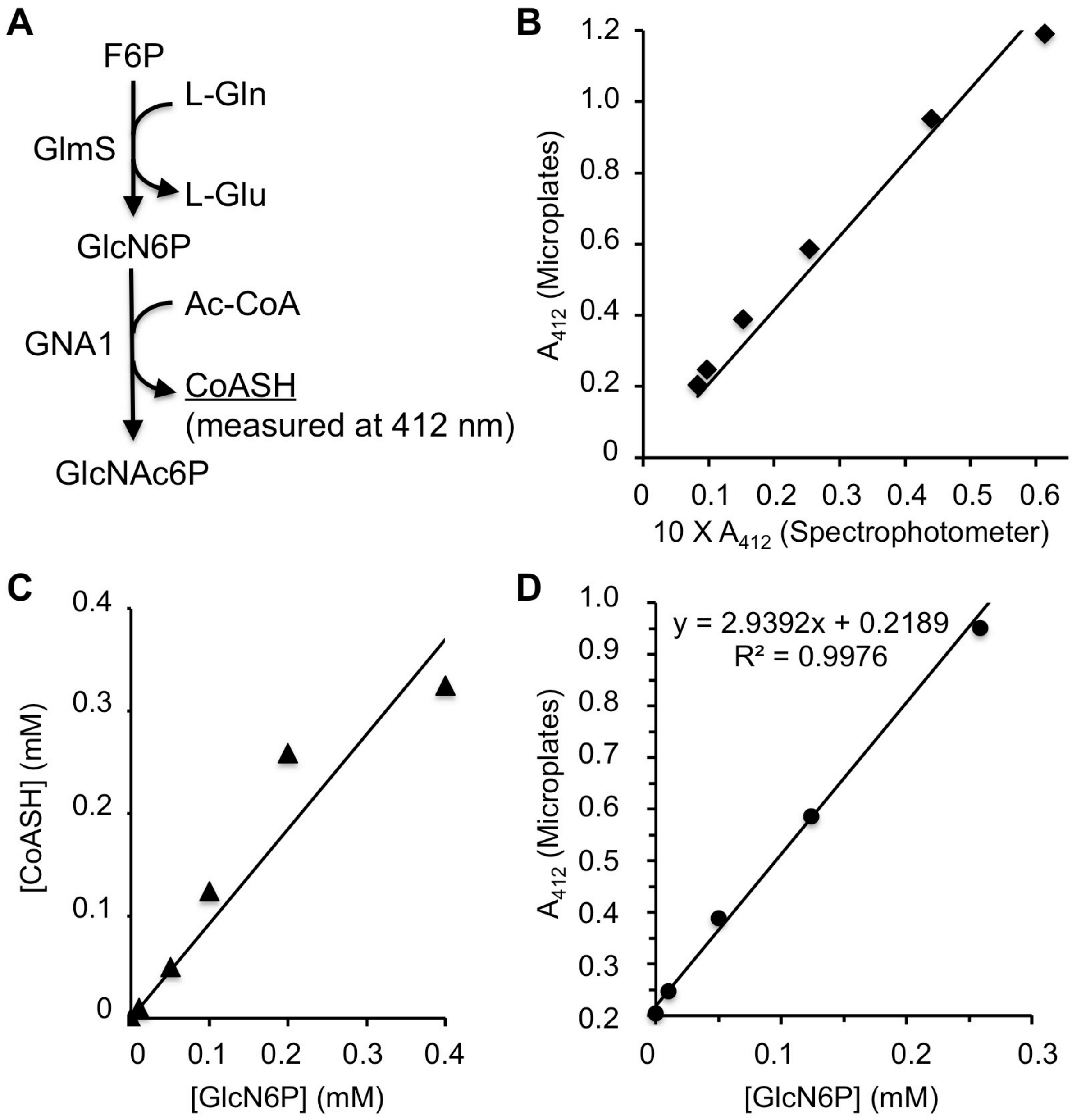

Figure S1: Characterization of GNA-1 activity by monitoring CoASH production.

A - Schematic representation of enzymatic-coupled assay using GNA-1 and monitoring the appearance of CoASH with the Ellman's reagent at 412 nm as described previously [1].

B - Graph of $A_{412\ nm}$ measured by the microplate reader versus $A_{412\ nm}$ measured by the spectrophotometer. We observed a linear correlation between the two sets of $A_{412\ nm}$.

To test GNA-1 activity and to calculate the amount of CoASH produced from GlcN6P, 50 mM Tris-HCl pH 7.5, 1 mM EDTA, 0 to 0.4 mM GlcN6P, 0.5 mM Ac-CoA, 0.5 mM DTNB were

pre-incubated as described previously [1]. The reaction was started by addition of 10 μg or 38 μg GNA1 in a final volume of 100 μl. The amount of CoASH was monitored at 412 nm by microplate reader at 37°C during 40 min. After 40 min, the $A_{412\ nm}$ of each sample, after a 10-fold dilution, was also measured by a spectrophotometer using a quartz cuvette with 1 cm of optical path (the extinction coefficient of TNB at 37°C ε_{412} = 13 800 M^{-1} cm^{-1}).

C - Graph of initial concentrations of substrate (GlcN6P) vs the final concentrations of product (CoASH) after 40 min of incubation with GNA-1. The concentrations of CoASH produced were calculated from $A_{412\ nm}$ measured with the spectrophotometer and using the extinction coefficient of TNB at 37°C (ε_{412} = 13 800 M^{-1} cm^{-1}). We observed a linear correlation between the initial concentrations of GlcN6P and the final concentrations of CoASH. In addition, we noticed that, in these experimental conditions, the enzymatic reaction is complete and all the substrate is converted into product. Here are presented the results obtained for 48 μg of GNA-1 but the same results were obtained with 10 μg of GNA-1 (data not shown).

D - Graph of initial concentrations of GlcN6P vs $A_{412\ nm}$ measured by microplate reader. We have observed a linear correlation between $A_{412\ nm}$ measured by the microplate reader and the concentration of GlcN6P. Consequently, it is possible to deduce a GlcN6P concentration from an $A_{412\ nm}$ measured by microplate reader in such experimental conditions; expressed in mM: [GlcN6P] = ($A_{412\ nm}$ -0.2189)/2.9392.

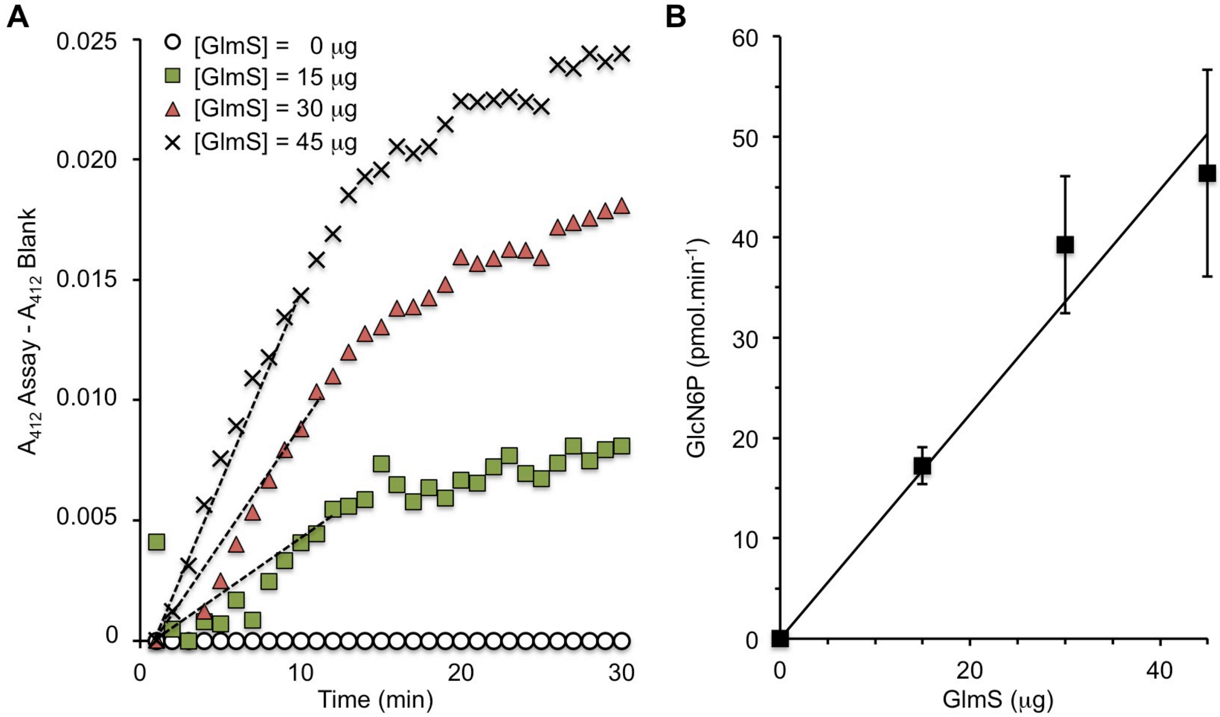

Figure S2: Kinetic of *B. subtilis* GlmS activity.

0, 15, 30 and 45 µg of GlmS were incubated in a final volume of 100 µl as indicated in the experimental procedures section.

A- The A_{412nm} was monitored during 30 min by a microplate reader at 37°C. For each experiment, the blank reaction without GlmS was used for background correction. Each curve is the average of 3 independent experiments. For each curve, we measured the difference of A_{412nm} after 10 min of reaction.

B- For each amount of GlmS, we calculated the number of pmoles of GlcN6P produced per min in a volume of 100 µl that corresponds to ($A_{412\ nm}$ /29.392)*100000. GlcN6P production is proportional to GlmS quantity.

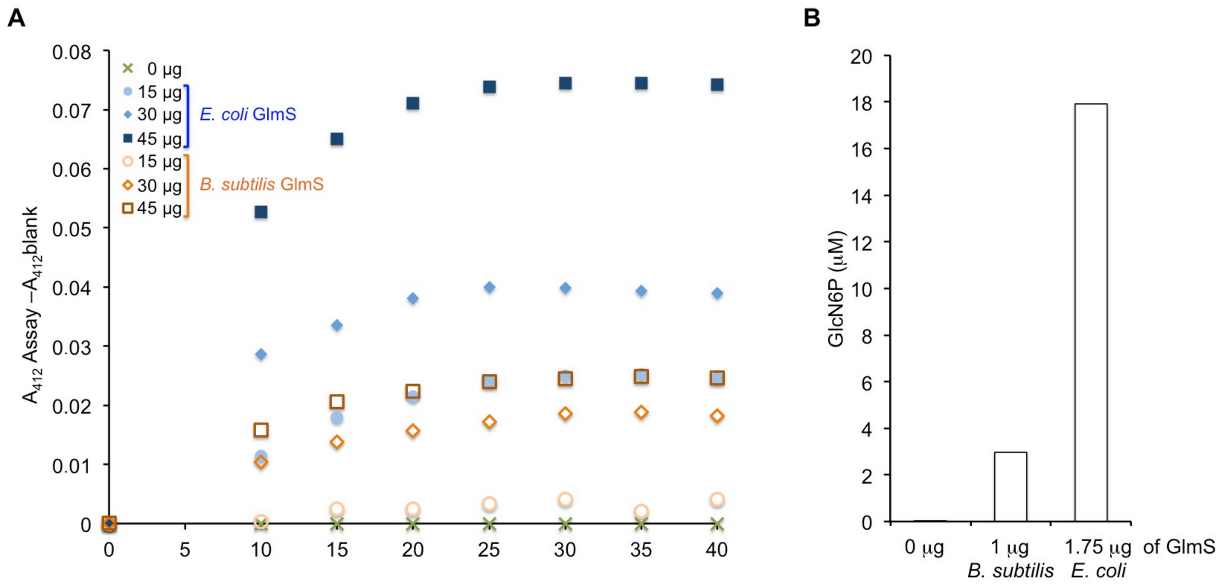

Figure S3: Comparison of the activity of GlmS from *B. subtilis* and from *E. coli*.

To produce the GlmS from *E. coli* with the same C-terminal 6His-tag, *glmS* gene was amplified by PCR from chromosomal DNA of *E. coli* MG1655 using specific primers and introduced into the pET21a (+) plasmid (Novagen) between the BamHI and XhoI sites, similarly that what we did for *B. subtilis glmS* gene. The obtained recombinant plasmids were introduced in *E. coli* NiCo21(DE3) to avoid contamination with untagged *E. coli* GlmS during purification on Ni-NTA column [2].

A- Comparison of GlmS from *E. coli* vs GlmS from *B. subtilis* by enzyme coupled assay. 0, 15, 30 and 45 μg of GlmS were incubated in a final volume of 100 μl as indicated in the experimental procedures section. The A_{412nm} was monitored during 40 min by a microplate reader at 37°C. For each experiment, the blank reaction without GlmS was used for background correction.

B- Comparison of GlmS from *E. coli* vs GlmS from *B. subtilis* by measuring direct production of GlcN6P with High Pressure Anion Exchange Chromatography coupled with Pulsed Amperometric Detection (HPAEC-PAD). GlmS was incubated at 37°C for 40 min in a buffer containing 47 mM KPO_4 (pH 7.2), 140 mM NaCl, 16 mM EDTA, 0.1 mM DTT, 2.3 mM L-Gln, 1.4 mM F6P. Then, 6.5 μl of the reaction mix were taken up and supplemented with 50 μl NaOH 0.5 M and 200 μl H_2O. GlcN6P quantified by HPAEC-PAD, using a Dionex ICS 3000 (Thermofisher, Waltham, MA). 25 μl of each sample were applied to a Dionex CarboPac PA1 column (4 x 250 mm) and the corresponding guard column (4 x 50 mm) at 30°C. Sugars were

eluted with the buffers 0.1 M NaOH and 0.5 M sodium acetate and 0.1 M NaOH as the eluents A and B, respectively. The following multi-step procedure was used: isocratic separation (0.5 min, 95 % A and 5 % B), non-linear (curved) separation gradient (6.5 min, 5 to 82.5 % B), column wash (2 min, 99 % B) and subsequent column equilibration (2.5 min, 95 % A and 5 % B). The flow rate was kept at 1 ml/min. Injection of samples containing Glucosamine-6P, Glucose-6P and Fructose 6-P (Sigma, Saint Louis, MO) at known concentrations (ranging from 4 to 100 μM) were used to identify and quantify the released phosphorylated sugars.

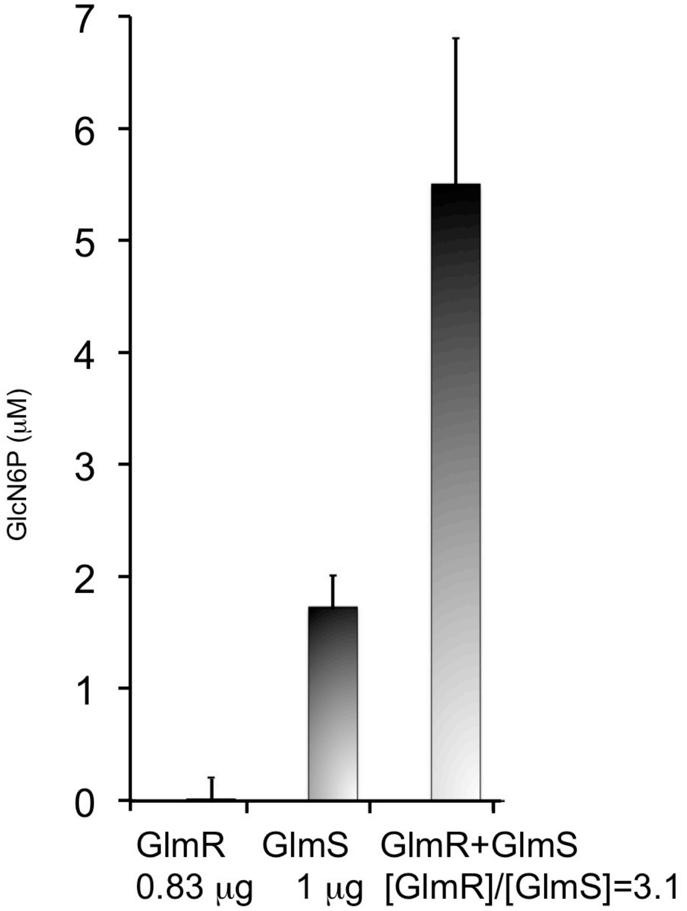

Figure S4: Stimulatory effect of GlmR on B. *subtilis* GlmS activity by measuring direct production of GlcN6P by HPAEC-PAD.

1 μg of *B. subtilis* GlmS was incubated at 37°C for 40 min in the absence or presence of 0.83 μg of GlmR (ratio [GlmR]/[GlmS] = 3.1) in a buffer containing 47 mM KPO4 (pH 7.2), 140 mM NaCl, 16 mM EDTA, 0.1 mM DTT, 2.3 mM L-Gln, 1.4 mM F6P. Then, 6.5 μl of the reaction mix was taken up and supplemented with 50 μl NaOH 0.5 M and 200 μl H_2O. GlcN6P quantified by HPAEC-PAD, using a Dionex ICS 3000 (Thermofisher, Waltham, MA) as indicated in the legend of Fig. S3B.

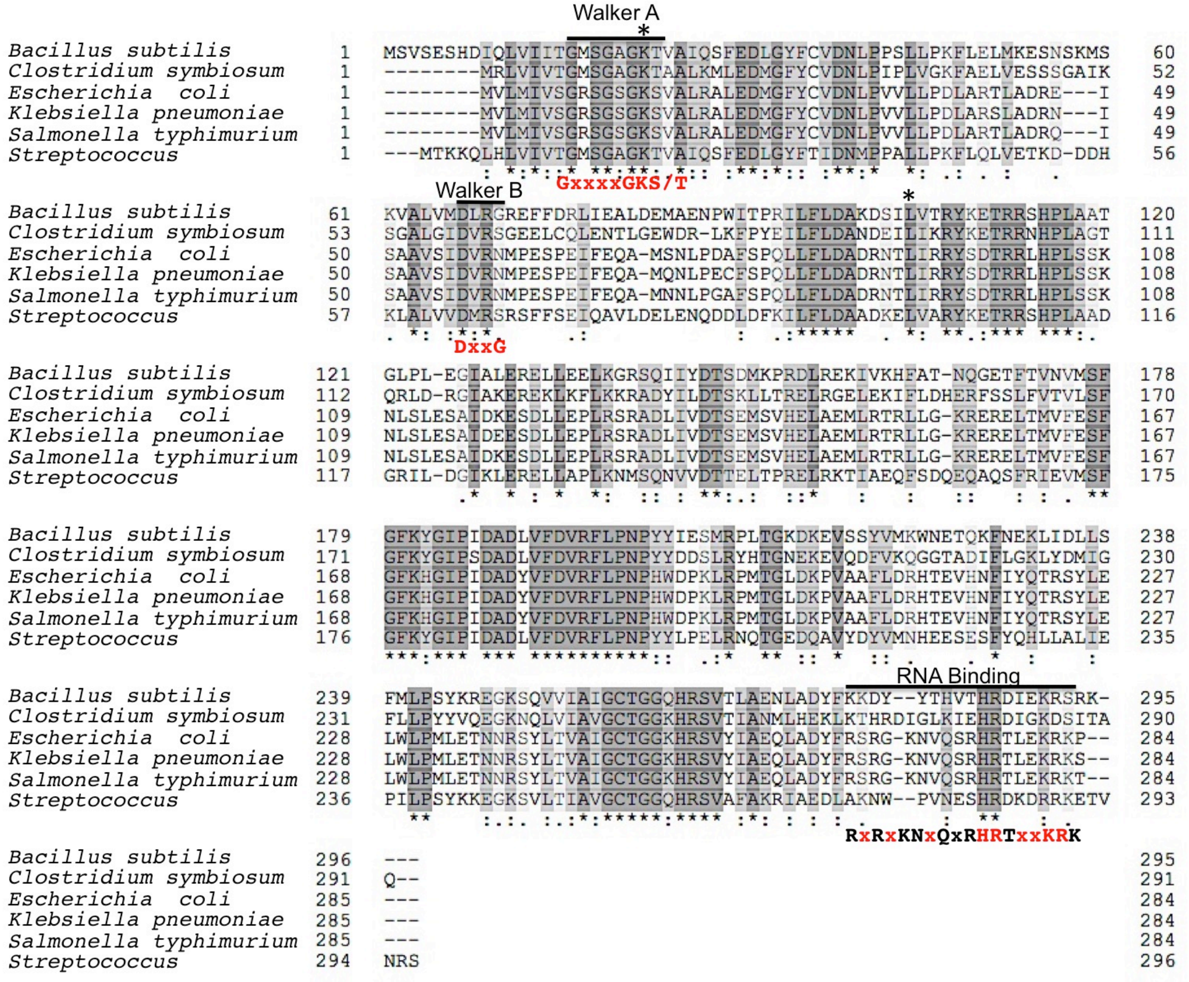

Figure S5: Sequence alignment of YvcJ/RapZ homologs.

Sequences were obtained from UniProt database and the alignment was performed with the Clustal Omega program (https://www.uniprot.org). The grey level reflects the similarity of amino acids: darker the grey, more the amino acid is conserved. The consensus sequences of Walker A and B motifs are indicated as well as the one present in the C-terminal part of RapZ from *E. coli* known to specifically bind to RNAs. In red are indicated the residues of the consensus sequences present in the sequence of YvcJ from *E.coli.* We observed that the consensus sequence present in the C-terminal part of RapZ from *E. coli* and known to specifically bind to RNAs is pretty well conserved in the sequences of proteins from *K. pneumoniae* and *S. typhimurium*. By contrast for proteins from *B. subtilis*, *C. symbiosum* and *Streptococcus*, the RNA-binding consensus is poorly conserved. The asterisks indicate the catalytic K22 and the L104 found to suppress growth defect of a *glmR* mutant [3].

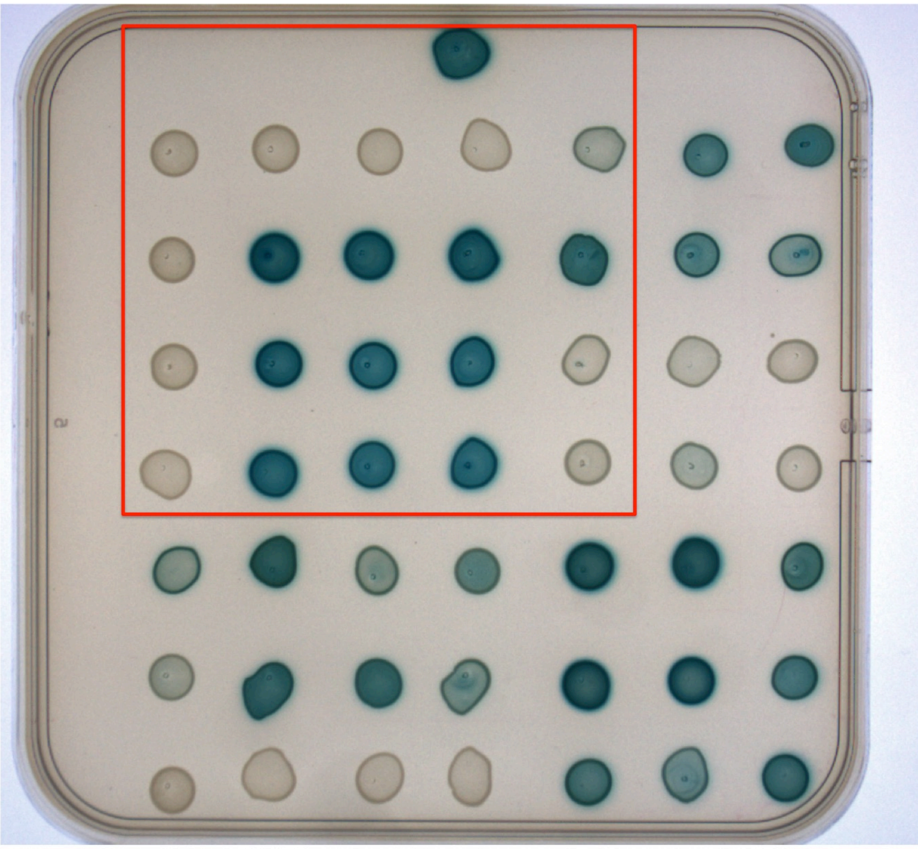

Figure S6: Original picture of the petri dish for bacterial two hybrid shown in Fig. 2B.

The part used for the Fig. 2B of the manuscript is in the red square

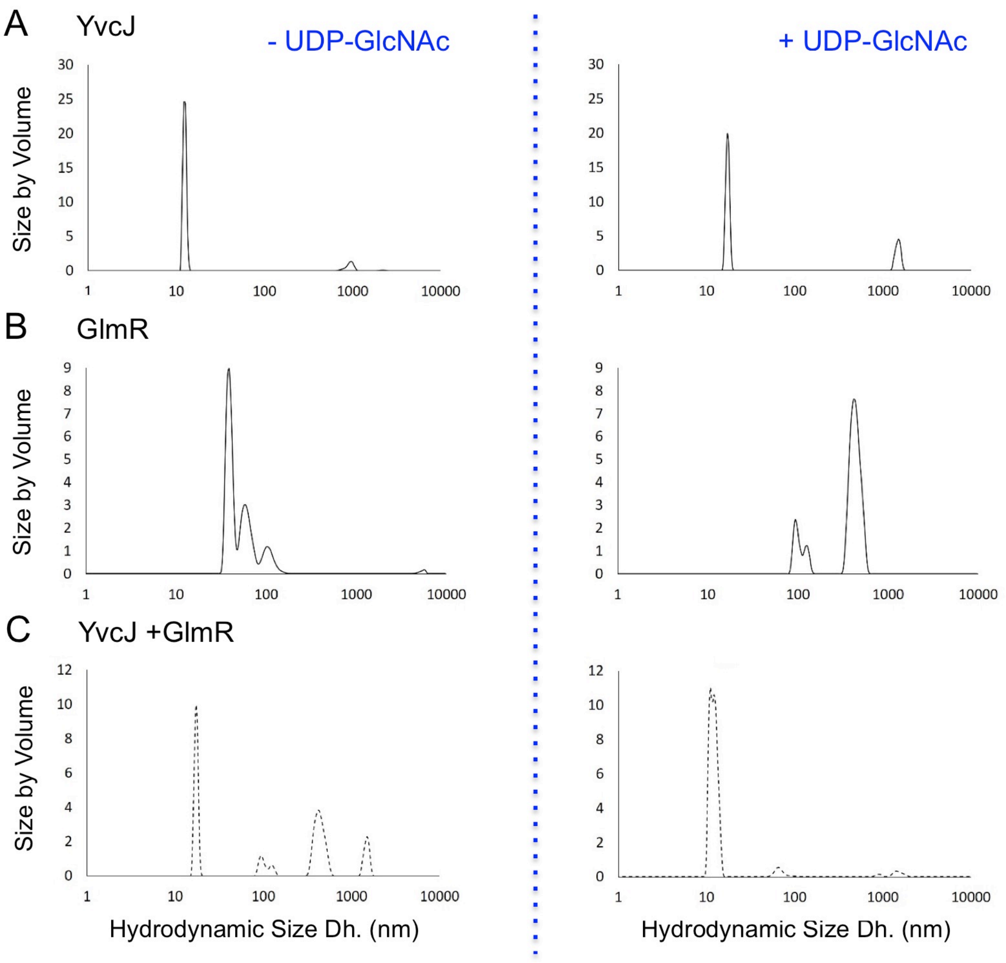

D

Sample	*D_h (nm)	Average Protein size in solution "Z.average" D (nm)
YvcJ	12,6± 0,404	700,7± 5,278
YvcJ with UDP-GlcNAc	17.37 ± 0.58	1241±47.35
GlmR	46,4±12,1	73,93 ± 0,73
GlmR with UDP-GlcNAc	409± 50,1	316 ± 1,102
YvcJ + GlmR	15,3± 3,46	197,1± 24,89
YvcJ +GlmR with UDP-GlcNAc	12.1±1.25	124,8 ± 34,77

Figure S7: Analysis of GlmR and YvcJ by DLS.

Volume weighted size distributions of YvcJ or GlmR alone or together in the absence (right panels) or the presence of 0.4 mM UDP-GlcNAc (left panels) at 25°C. All experiments were carried out in triplicate.

A- YvcJ 24μM.

B- GlmR 7μM.

C- YvcJ 24μM + GlmR 7 μM.

D- Size distribution shown in hydrodynamic diameter* of the major peak using volume weighted distribution and the average protein size in solution using intensity weighted distribution. Three independent measurements were performed.

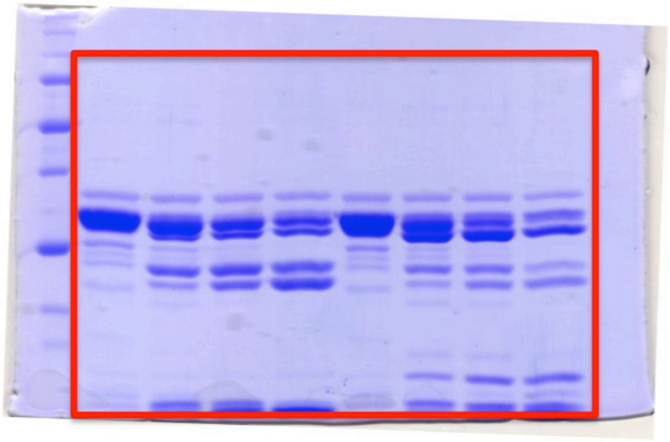

Figure S8: Original picture of the full-length gel used for Fig. 5A.

The part used for the Fig. 5A of the manuscript is in the red square

REFERENCES

1 Li, Y. *et al.* An enzyme-coupled assay for amidotransferase activity of glucosamine-6-phosphate synthase. *Anal Biochem* **370**, 142-146, doi:10.1016/j.ab.2007.07.031 (2007).

2 Robichon, C., Luo, J., Causey, T. B., Benner, J. S. & Samuelson, J. C. Engineering *Escherichia coli* BL21(DE3) derivative strains to minimize *E. coli* protein contamination after purification by immobilized metal affinity chromatography. *Appl Environ Microbiol* **77**, 4634-4646, doi:10.1128/AEM.00119-11 (2011).

3 Patel, V., Wu, Q., Chandrangsu, P. & Helmann, J. D. A metabolic checkpoint protein GlmR is important for diverting carbon into peptidoglycan biosynthesis in *Bacillus subtilis*. *PLoS Genet* **14**, e1007689, doi:10.1371/journal.pgen.1007689 (2018).
